# The Use of Traditional Chinese Medicine Among Chinese Seniors in Canada and the United States: A Scoping Review

**DOI:** 10.3390/healthcare14101310

**Published:** 2026-05-12

**Authors:** Ping Zou, Yanjin Huang, Tingqi Huang, Daniel D’Souza, Xiyi Wang, Hui Zhang, Yeqin Yang, Yan Luo, Yao Wang

**Affiliations:** 1 School of Nursing, Nipissing University, 100 College Drive, North Bay, ON P1B 8L7, Canada; 2 School of Nursing, Ningxia Medical University, 1160 Shengli Street, Yinchuan 750004, China; 3 Faculty of Health Sciences, McMaster University, 1280 Main St. W, Hamilton, ON L8S 4L8, Canada; 4 Temerty Faculty of Medicine, University of Toronto, 27 King’s College Cir, Toronto, ON M5S 1A1, Canada; danielm.dsouza@mail.utoronto.ca; 5 School of Nursing, Shanghai Jiao Tong University, Shanghai 200025, China; 6 Department of Nursing, Guizhou Provincial People’s Hospital, Guiyang 550002, China; 7 School of Nursing, Zhejiang Chinese Medical University, 548 Binwen Road, Binjiang District, Hangzhou 310053, China; 8 Faculty of Nursing, Health Science Center, Xi’an Jiaotong University, No. 76 Yanta West Road, Xi’an 710061, China; 9 Xiang Ya School of Nursing, Central South University, Changsha 410013, China

**Keywords:** use of Traditional Chinese Medicine, older adults, seniors, Chinese, immigrants, scoping review

## Abstract

**Introduction**: Chinese seniors in North America represent a growing population, and Traditional Chinese medicine (TCM) continues to play an important role in their health practices; however, TCM use has not been comprehensively synthesized from an immigrant perspective in North America. The purpose of this scoping review is to explore the use of TCM by Chinese seniors in Canada and the United States of America (USA). **Methods**: This scoping review is written in accordance with PRISMA guidelines. PubMed, PsycINFO, CINAHL, AgeLine, ERIC, ProQuest, Nursing and Allied Health Database, PsycARTICLES, Sociology Database, and Education Research Complete were selected for the literature search, which was conducted in August 2025. Articles were included if they investigated the use of any form of TCM among Chinese seniors aged 65 years or older living in the USA and Canada, and were published in an English-language peer-reviewed journal. **Results**: Twenty-four studies were included in this review, with a total sample size of 7288. The findings indicated that, in the majority of the included studies, over half of the Chinese seniors used some form of TCM. TCM therapies included over-the-counter herbal products, TCM-based physical activities, TCM practitioner consulting, and TCM food practices and therapies. Use of TCM among Chinese seniors was related to musculoskeletal symptoms, cardiovascular symptoms, mental health, severe acute respiratory syndrome prevention, cancer screening, and oral health. Chinese seniors tend to integrate TCM with Western medicine in their healthcare practices. Several factors from individual, familial, and community levels influenced Chinese seniors’ use of TCM. **Conclusions**: Future research should investigate the integration of TCM with Western healthcare, the training of healthcare providers to improve their understanding of TCM, and the underlying mechanisms of TCM products.

## 1. Introduction

Traditional Chinese Medicine (TCM) has been developed and practiced in China for over 2000 years, encompassing a range of techniques, including herbal medicine, acupuncture, massage therapy, mind–body exercise, and dietary recommendations. TCM takes a holistic approach to addressing how illnesses manifest in the body and treats the whole patient rather than a specific disorder [1]. In the United States of America (USA), TCM falls under the umbrella term of “complementary and alternative medicine”, which is defined by the National Institutes of Health as healthcare outside of mainstream or conventional Western medicine [2]. Although unconventional, Chinese herbal products have been investigated for medical problems, such as stroke, heart diseases, mental disorders, and respiratory diseases. Approximately one in five Americans uses herbal products [3].

In Canada, acupuncture and TCM are not regulated on the federal level [4]. However, five provinces (British Columbia, Ontario, Alberta, Quebec, and Newfoundland and Labrador) have established provincial laws to protect the public’s right to safe, competent, and ethical services offered by registered TCM practitioners, TCM acupuncturists, and/or TCM herbalists who are members of the regulatory bodies [4]. In Ontario, Canada, the practice of TCM is regulated by the College of Traditional Chinese Medicine Practitioners and Acupuncturists of Ontario [5]. The TCM field is a self-governing profession that can set standards of practice and entry-to-practice requirements [5]. In 2016, more than three-quarters of Canadians (79%) had used at least one complementary or alternative therapy sometime in their lives [6].

Chinese Americans are the largest Asian-origin group in the U.S., making up 24% of the Asian population, or 5.4 million people [7]. Thirteen percent, or 702,000, of Chinese Americans are 65 years of age or older [8]. Additionally, by the middle of the century, Asian Americans are projected to be the nation’s largest immigrant group [7]. In 2016, there were 1.8 million Chinese-identifying individuals in Canada [9]. According to 2011 figures, 138,000 adults in Canada aged 65 and above identify as Chinese, accounting for 10.5% of the total Chinese-Canadian population [10]. Over the next 20 years, Canada’s senior population (65 years or older) is expected to grow by 68% [11]. In both Canada and the USA, there is a large and aging population of Chinese immigrants, which warrants the attention of healthcare providers and policymakers to provide culturally sensitive healthcare.

Canada and the United States provide important and comparable contexts for examining the use of TCM among Chinese senior immigrants [1,2,3]. Both countries have long histories of Chinese immigration and share Western biomedical healthcare systems within which TCM is positioned as a complementary or alternative approach and is formally recognized and regulated in certain regions, such as Canada [2,4,5,6]. Chinese populations represent one of the largest and fastest-growing immigrant groups in North America [7,8,9], and older adults within these communities often experience challenges related to language barriers, cultural continuity, and access to culturally appropriate healthcare services [10,11]. Examining the use of TCM in this specific geographical context allows for a more focused understanding of how cultural health practices are maintained, adapted, or negotiated within Western healthcare environments [2,6].

The relationship between TCM and Western medical practices calls for further examination. Cultural views of health may affect the care needs of aging Chinese seniors. Throughout the history of China, the population has been influenced by the teachings of Confucianism and Daoism, which have shaped the way in which health, illnesses, and treatment are conceptualized and practiced. Confucianism emphasizes harmony, filial piety, and the moral responsibility to maintain one’s health, which may encourage older adults to engage in preventive practices and seek treatments that align with family values and social expectations. Daoism, with its focus on balance, naturalness, and the flow of energy, supports holistic approaches to health and may influence the preference for TCM therapies that aim to restore equilibrium rather than treat isolated symptoms. Together, these philosophies may shape decision-making by promoting integrative health practices and influencing how Chinese seniors navigate between TCM and Western medicine [12]. It is important that Western healthcare providers practice culturally sensitive healthcare with Chinese seniors to facilitate patient-centered care. Many TCM users do not disclose their use to their Western physicians, which raises concerns, as there may be side effects due to interactions between Chinese and Western medications [3,13,14]. Chinese seniors may prefer to cope with the symptoms themselves or use emergency services, meaning that they often deal with symptoms until they are unmanageable [15]. Since Canadian and USA Chinese seniors likely address their health through both TCM and Western medicine, addressing their integration may improve the physician–patient relationship and the care that these patients receive [16]. Therefore, it is vital that the use of TCM among Chinese seniors is explored, and the effects of TCM are considered in a geriatric medicine setting.

Existing reviews have synthesized information on the use of TCM in Western contexts; however, these reviews have not focused on Chinese immigrant populations in North America, particularly older adults. A 1994 literature review broadly defined TCM and emphasized potential clinical implications, such as herb–drug interactions [14]. A 2014 systematic review summarized commonly used herbal medicines among older adults globally and highlighted the importance of patient–provider communication regarding supplement use [13]. A 2021 systematic review examined the effects of Tai Chi interventions and reported improvements in blood pressure among patients with hypertension [17]. While these reviews provide valuable insights into specific aspects of TCM use, they focus on general populations, specific therapies, or clinical outcomes, and do not address the experiences of Chinese immigrant seniors within the North American healthcare context. To date, no review has comprehensively examined the use of TCM among Chinese seniors in North America from an immigrant perspective, incorporating cultural, behavioral, and healthcare system factors. This scoping review aims to map the existing literature on the use of TCM among Chinese senior immigrants in Canada and the United States, including patterns of use, influencing factors, and associated health contexts. The research questions are: (1) What are the experiences of using various types of TCM therapies among Chinese seniors in Canada and the United States, and (2) What factors are influencing the use of TCM therapies?

## 2. Methods

This study employed a scoping review methodology to map the breadth and nature of the existing literature on the use of TCM among Chinese seniors in Canada and the United States. A scoping review was considered appropriate given the heterogeneity of study designs, populations, and contexts in this field. Unlike systematic reviews, which typically address narrowly defined questions and focus on synthesizing homogeneous evidence, scoping reviews are particularly useful for exploring complex and under-researched topics, identifying key themes, and highlighting research gaps. The protocol and reporting of results of this scoping review were based on the PRISMA scoping review statement [18]. This Scoping Review has been registered on https://osf.io/sdkb3/overview (accessed on 29 April 2026).

### 2.1. Eligibility Criteria

Studies were included if they had investigated the use of any form of TCM by Chinese seniors living in the USA and Canada and were published in an English-language, peer-reviewed journal. Only English-language, peer-reviewed studies were included, which may introduce publication bias. Seniors were defined as individuals aged 65 years and above, as this threshold is widely recognized in gerontological research and policies in North America. The age of 65 is commonly used to define older adulthood in epidemiological studies and aligns with eligibility criteria for social programs such as public pensions and healthcare in North America. Using this threshold enhances consistency with the existing literature and facilitates comparison across studies examining health behaviors and healthcare utilization among older populations. In addition, this definition reflects a life stage often associated with retirement, increased healthcare needs, and age-related changes in health status, making it appropriate for examining patterns of TCM use among older adults. Therefore, we included studies in which the mean age of participants was 65 years or older. In addition, studies were included if at least 50% of their participants were Chinese senior immigrants from Canada or the United States. This criterion was applied to ensure sufficient inclusion of relevant studies in a field where research exclusively focused on older adults is limited. Forms of TCM included herbal medicines, Tai Chi, Qi Gong, and acupuncture. We included quantitative, qualitative, and mixed-method studies in this review.

Studies were excluded if they: (a) had a study population whose mean age was lower than 65 years old; (b) had a sample whose majority was not Chinese; (c) discussed non-TCM interventions, (d) were protocols for a future study, (e) did not have an author, or (f) did not have an accessible electronic text document.

### 2.2. Information Sources

Various health-related, psychological, sociological, and educational science databases, including PubMed, PsycINFO, CINAHL, AgeLine, ERIC, ProQuest, Nursing and Allied Health Database, PsycARTICLES, Sociology Database, and Education Research Complete, were selected for the literature search. Google Scholar was also used to identify any potential articles that may have been missed by the aforementioned databases, to ensure the utmost inclusivity of articles.

### 2.3. Search Strategy and Selection of Evidence

**Figure 1:**
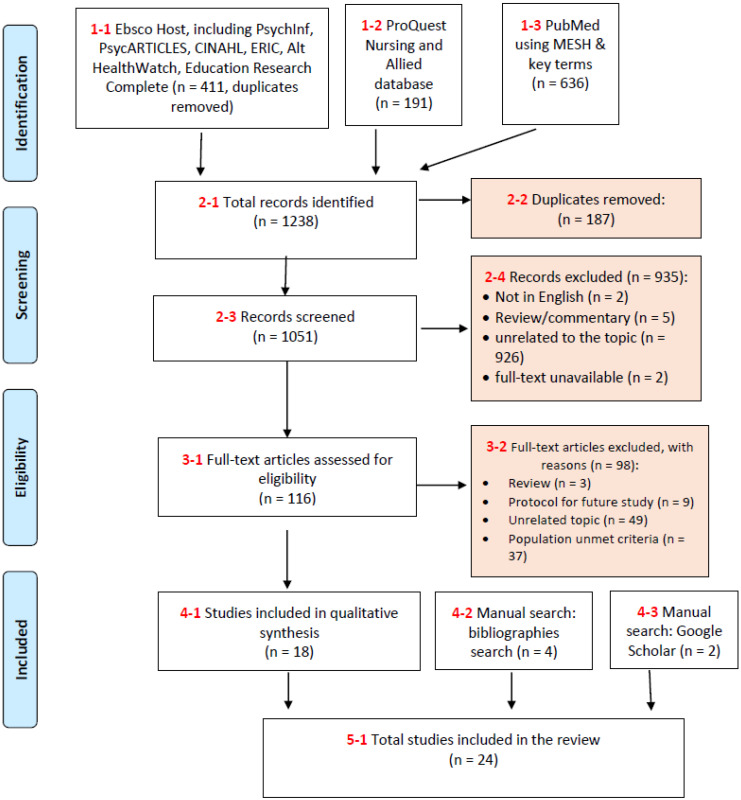
Study selection flow diagram—TCM use among Chinese seniors in North America.

The search was conducted by three researchers (DD, TH, and PZ, Figure 1). This scoping review was guided by the Population–Concept–Context (PCC) framework. The population of interest was Chinese seniors, the concept was the use of TCM, and the context was Canada and the United States. The databases were systematically searched by title/abstract using a combination of the keywords (aged OR elder* OR old* OR senior* OR geriatric*) AND (tradition* Chinese medicine OR Chinese tradition* medicine OR acupuncture OR tai chi OR qi gong OR herb*) AND (North America* OR United States OR Canada OR Canadian* OR America* OR USA). In addition, the MeSH terms used for PubMed were ((“Aged”[Mesh]) AND “Medicine, Chinese Traditional”[Mesh]) AND “North America”[Mesh]. The citations were exported into EndNote to remove any duplicates. The titles and abstracts of all citations were screened for relevance based on the established eligibility criteria. All eligible articles were searched for full-text documents, and the full-text documents were carefully reviewed, with reasons for exclusion noted. Furthermore, tables of contents of key journals were hand searched for the past two years, and the reference lists of all eligible articles were manually searched for additional titles not returned in the initial search. The literature search was conducted in August 2025.

### 2.4. Quality Assessment

The Critical Appraisal Skills Programme (CASP) checklists and the National Heart, Lung, and Blood Institute (NHLBI) Quality Assessment Tool for Observational Cohort and Cross-Sectional Studies were used to assess the methodological quality of included studies [19,20,21]. CASP checklists were applied to qualitative and cohort studies, while the NHLBI tool was used for cross-sectional studies. These tools are not designed to generate a single quantitative score but rather to support a comprehensive evaluation of methodological rigor, including study design, data collection, analysis, and reporting. Based on these criteria, studies were categorized as low, moderate, or high quality (CASP), or as poor, fair, or good (NHLBI). Three researchers (TH, DD, and PZ) independently assessed each study, and discrepancies were resolved through discussion and consensus. Based on the quality assessment, qualitative studies were rated as moderate to high (on a scale including low, medium, and high), all cohort studies were rated as high (on a scale including low, medium, and high), and all cross-sectional studies were rated as good (on a scale including poor, fair, and good). Studies rated as low quality were to be excluded; however, no studies were rated as low quality and, therefore, no studies were excluded on this basis (see Supplementary Tables S1–S3). Quality appraisal informed the interpretation of findings during the narrative synthesis.

### 2.5. Data Extraction

Data were independently extracted by three reviewers (TH, DD, and PZ) based on pre-determined criteria. From each article, various data, including authors, year of publication, study population, research design, recruitment method, sample size, sample characteristics, comparison group, features of the intervention, outcomes, measurements, significant findings, limitations, and future direction were extracted. The data were collected and organized into an Excel spreadsheet. The reviewers discussed disagreements in data extraction until consensus was reached.

### 2.6. Synthesis of Results

Once the data were organized in Excel, descriptive statistics were used to present the characteristics of included studies. Thematic analysis was then used to summarize the findings of each research question. Categorized results were compared among reviewers (TH and PZ), and any disagreements were resolved through consensus. Due to the heterogeneity of the measurement tools used by the included studies, a meta-analysis was not performed since attempting to combine different measurements for the same variable would be inappropriate.

## 3. Results

### 3.1. Characteristics of Included Papers

Twenty-four published papers were included in this review (Table 1). Eighteen (75%, 18/24) studies were conducted in the USA, five (21%, 5/24) in Canada, and one (4%, 1/24) study was conducted with participants from Canada and Australia. Twenty-three (96%, 23/24) papers were solely based in the community, and one (4%, 1/24) was based in the clinical setting.

Eight (33%, 8/24) studies used a qualitative methodology, with five (21%, 5/24) studies utilizing an interview format, and three (13%, 3/24) studies utilizing a focus group. Fifteen (58%, 14/24) used a quantitative methodology, with twelve (50%, 12/24) cross-sectional studies and three (13%, 3/24) quasi-experimental cohort studies. There was one (4%, 1/24) mixed-methods study utilizing interviews and a constant comparison method of quantitative values.

The total unique sample size across the 24 included studies was 7346 participants after accounting for overlapping study populations. Several studies were based on the same underlying datasets. Specifically, four articles reported on the same Chicago cross-sectional study, two articles reported on the same Canadian survey, and three articles reported on the same San Francisco intervention study. Although these studies examined different research questions and outcomes, each dataset was counted only once in the total sample size calculation to avoid duplication.

Overall, the findings synthesized in this review are supported by studies of consistently moderate to high methodological quality. Qualitative studies were rated as moderate to high, cohort studies as high, and cross-sectional studies as good. No studies were rated as low or poor.

### 3.2. Experiences of Using Various Types of TCM

#### 3.2.1. Prevalence

Three (13%, 3/24) studies reported on the prevalence of TCM use in Canada [23,26,34]. In 1998, in Vancouver and Victoria, British Columbia, amongst 602 Chinese seniors, approximately 50% engaged in TCM [23]. In 2002, in the Kitchener–Waterloo Region of Ontario, among 106 Chinese seniors, 50% reported using TCM [26]. In a 2007 study regarding seven regions: Victoria, Greater Vancouver, Calgary, Edmonton, Winnipeg, Greater Toronto, and Greater Montreal, among 2167 Chinese senior immigrants, over 65.4% reported using TCM [34]. Among these 2167 Chinese seniors, over-the-counter Chinese herbs were used by 50.7% of participants, followed by over-the-counter Chinese herbal formulas (41.9%), services of Chinese TCM herbalist practitioners (24.1%), acupuncturists (8.3%), bone setters (4.9%), moxibustion specialists (2.3%), and Qi Qong specialists (0.7%) [34].

Two (8%, 2/24) studies reported on the use of TCM in the USA [28,41]. In 2004, in Boston, Massachusetts, among 177 older Chinese Americans, 26% indicated that they visited traditional Chinese doctors at least once in the past year [28]. In 2015, in Chicago, Illinois, among 3158 older Chinese Americans, 76% reported TCM use in the past year [41]. Regarding TCM use, 39.9% used over-the-counter topical herbal products, 35.5% used over-the-counter oral herbal products, 12.4% used Tai Chi, 12.3% used massage therapy, 11.8% used acupuncture, 10.8% used prescribed oral herbal products, 9.1% used prescribed topical herbal products, and 23.4% used another form of TCM [41].

#### 3.2.2. Health Beliefs and Cultural Meanings of TCM

Five (21%, 5/24) studies presented TCM health beliefs and cultural meanings [24,25,36,37,39]. Incorporating TCM into Chinese seniors’ health practices allowed them to perform and reaffirm their cultural identity as Chinese, fulfill social roles, and pass down cultural heritage [39]. The Hot and Cold Theory is an important concept of TCM [24,25]. Cold pertains to the quality of certain TCM therapies that have the capacity to slow down movement and bring more stability [25]. Whereas Hot TCM therapies facilitate movement and increase activity [25]. Chinese seniors viewed the balance of Hot and Cold as essential for their well-being [24,25].

TCM constitution refers to the natural state of an individual based on physiological functions and psychological conditions [24]. It can be further divided into normal constitution and unbalanced ones [24]. Every individual has their own unique body constitution, which changes over time and can make one susceptible to certain diseases [24]. For example, someone with a hot, unbalanced constitution could be characterized by a preference for cold foods [24]. To appeal to one’s natural state of constitution, individuals can determine their constitution and consume foods that are inherently hot or cold to maintain balance [24,25,36].

#### 3.2.3. Use of Various Types of TCM Therapies

Chinese seniors used various types of TCM therapies, including over-the-counter herbal products, TCM-based physical activities, consultations with TCM practitioners, and TCM food practices and therapies. Seventeen (71%, 17/24) of the studies explored over-the-counter herbal products, which included herbal teas and medicines [23,25,26,27,29,30,34,35,36,37,38,39,40,41,42,43,44]. Herbal teas mentioned included ginseng, tang-gui, and ju-hao [25]. Currently, Beijing Royal Jelly Natural Dietetics is a popular herbal medication used in the maintenance of health [25]. Three (13%, 3/24) of the studies found that over-the-counter herbal products were the most frequently used type of TCM among Chinese senior immigrants in North America [25,34,41].

Nine (38%, 9/24) studies reported TCM-based physical activities, such as Tai Chi, Tai Ji Quan, or Qi Gong [25,27,31,32,33,34,36,43,44]. Chinese elders believe that Tai Ji Quan and Qi Gong are useful for promoting the circulation of Qi and blood throughout the body [25]. Studies reported improvements in aerobic endurance, blood pressure, and mood states in relation to Tai Chi interventions [31,32,33].

Eight (33%, 8/24) studies explored consultations with TCM practitioners, which included seeing specialists in acupuncture, massage therapy, cupping, bone setting, herbalist, Qi Gong, or moxibustion [23,27,29,30,34,41,43,44]. Participants consulted both Western and TCM practitioners for their health [23,27]. One qualitative study found that men are more likely to consult both Western and TCM practitioners but rely on Western physicians for final healthcare decisions [27].

Five (21%, 5/24) studies reported on TCM food practices and therapies [23,24,36,39,40]. Eating and drinking traditional Chinese foods could balance the body and treat minor or serious illnesses [23,36,39]. For example, to strengthen one’s immune system during the SARS virus outbreak, Chinese elders utilized food therapy to recommend cold foods that balance against the hot environments where the virus existed [36].

#### 3.2.4. Use of TCM in Various Illnesses and Related Outcomes

This section summarizes outcomes reported in the included studies and does not evaluate the effectiveness of TCM interventions. Five (21%, 5/24) studies reported on the use of TCM to treat musculoskeletal symptoms or pain [24,27,32,39,42]. A Los Angeles qualitative study reported the use of TCM ointments for arthritis, and acupuncture for lower back pain [27]. One Chicago cross-sectional study reported that seniors with musculoskeletal symptoms have greater overall TCM utilization (OR = 2.10, 95% CI = [1.76, 2.52]), specifically in massage therapy (OR = 3.41, 95% CI = [2.51, 4.63]), herbal medicine use (OR = 2.68, 95% CI = [2.28, 3.14]), and acupuncture use (OR = 2.49, 95% CI = [1.87, 3.32]) [42]. In terms of the association between Tai Chi and musculoskeletal symptoms, there were no statistically significant findings (OR = 1.18, 95% CI = [0.93, 1.50]) [42]. After a 12-week Tai Chi intervention, statistically significant (*p* value < 0.05) improvements were found in balance, muscular strength, endurance, and flexibility measures in the Chinese adult population [32].

Four (17%, 4/24) articles reported on TCM and cardiovascular symptoms [31,32,33,35]. Three articles reported on a San Francisco quasi-experimental intervention study for Chinese adults with cardiovascular disease risk [31,32,33]. The community-based 60 min Tai Chi intervention was conducted twice a week, for twelve weeks [33]. A Tai Chi instructor proficient in the Yang-Style 24-posture taught the sessions [31]. The Tai Chi intervention study reported statistically significant (*p* value < 0.05) improvements in aerobic endurance and reductions in blood pressure at rest [33], and statistically significant (*p* value < 0.05) improvements in perceived stress and mood states [31]. As well, statistically significant (*p* value < 0.05) improvements were reported in balance, muscular strength, endurance, and flexibility measures after the intervention [32]. Regarding hypertension medication adherence and TCM use, one USA cross-sectional study reported that individuals utilizing TCM for hypertension treatment had lower adherence to Western medication [30].

Other health conditions, such as severe acute respiratory syndrome (SARS), cancer prevention, and oral health, also relate to the use of TCM among Chinese seniors in Canada and the USA. One (4%, 1/24) qualitative study investigated the use of TCM by seniors in Edmonton, Canada, to prevent SARS [36]. The use of TCM was mainly preventative in nature, including the use of herbs, special foods, Qi Gong, and Tai Chi [36]. Participants believed Tai Chi and Qi Gong boosted one’s immune system against SARS [36]. One (4%, 1/24) cross-sectional study examined female American Chinese seniors’ cancer screening behaviors and use of TCM [43]. The study found that those who had received breast cancer screening were more likely to use acupuncture, massage therapy, and Tai Chi [43]. One (4%, 1/24) qualitative study investigated the use of TCM by seniors in Canada and Australia to maintain oral health [40]. Herbs and specific foods were consumed for the prevention and treatment of oral diseases [40]. Herbal teas included honeysuckle flower, chrysanthemum, and tarragon to decrease inflammation [40]. To treat swollen and infected gums, participants used herbs such as Xia Sang Ju or food therapy such as “24 Tastes”, with rice porridge [40].

#### 3.2.5. Integration of TCM with Western Medicine

In eight (33%, 8/24) studies, participants reflected on the use of TCM and Western medicine [25,26,27,29,36,38,39,43]. Chinese immigrant seniors prefer TCM for the daily management of chronic illnesses [25,27,29,38,39]. Participants believe TCM is the best practice for addressing the root cause of chronic diseases, as it is slower acting and more natural in comparison to Western medicine [25,27,29,38,39]. Nonetheless, participants prefer Western medical specialists for treating acute diseases when immediate results are necessary [25,27,29,38,39,40]. One participant shared the importance of using Western healthcare for diagnosis and acute treatment [38]. Western medicine allowed for the accurate diagnosis of cancer, and further diagnosis of polyps, and the surgeon immediately removed the tumors [38]. If he had used TCM, the participant would have undergone treatments that may not have addressed the tumors directly [38].

Participants use both Western and TCM together when they see fit [25,27,29,38,39,40]. In a cross-sectional study of 106 seniors, 21.7% used both TCM and Western medications together [26]. Chinese seniors will attempt to treat illnesses with TCM first, consult friends and family, and, as a last resort, seek a Western medicine physician [27,39]. For example, in treating arthritis, elders will consult with Western physicians while also using Chinese herbs and ointments [27]. In a pandemic setting, senior immigrants used both Western and TCM to prevent SARS infection, based on recommendations of various Western and Eastern health authorities [36].

#### 3.2.6. Factors Influencing the Use of TCM Therapies

Individual, family, and community factors can influence the use of TCM therapies. Nineteen (80%, 19/24) studies reported on individual factors, including physical health, mental health, and others [22,23,24,25,26,27,28,29,34,35,36,37,38,39,40,41,42,43,44]. In terms of physical health, Chinese seniors in the USA and Canada who experience more physical symptoms of disease are more likely to use TCM [22,23,25,26,27,34,39,41,42]. Canadian seniors who experience pain symptoms are ten times more likely to use TCM [26].

The impact of mental health on the use of TCM is conflicting. One Canadian study found that mental health was associated with a greater likelihood of TCM use [34]. While another USA study found that acupuncture and massage therapy were more frequently used by Chinese American seniors with depression symptoms [44]. In opposition, one cross-sectional study reported that anxiety or depression did not have an impact on TCM use [41].

Other individual factors include age, gender, language, individual beliefs, and education. Firstly, the included articles reported conflicting findings regarding age. Three (13%, 3/24) quantitative studies reported that younger participants were more likely to use TCM [23,37,41], all of which were rated as good quality, whereas one cross-sectional study, also rated as good quality, found that Chinese seniors were more likely to use TCM [44]. Secondly, women were reported to be more likely to use TCM [27,35]. Thirdly, language was identified as a facilitator of TCM use, as seniors were able to communicate with Chinese healthcare providers in their native language [27,29]. Lastly, individuals in the USA and Canada who held stronger beliefs in traditional Chinese practices were more likely to use TCM [23,25,34,39], and some did not view Western medical services as part of their regular care [39]. Findings related to education were also inconsistent. One study, rated as good quality, found that participants in the USA with higher education were more likely to use TCM [41], whereas another study, rated as good quality, reported that older Chinese individuals in Canada with lower education levels were more likely to agree with the use of TCM [37].

Four (17%, 4/24) studies reported that family factors influence the use of TCM [25,36,39,40]. Family factors included filial piety and family history. Filial piety can be demonstrated through service to others, whether it is family or community members [36]. Firstly, American and Canadian family members act as facilitators to the practice of TCM, as family members practice filial piety to the seniors, and place importance on helping them maintain their health through their choice of either TCM or Western medicine [36,39]. In addition, Chinese seniors viewed themselves as caregivers for the whole family [39]. To them, the meaning of life in a foreign land was based on what they could do for their family [39]. The ease of use and availability of TCM gave them the ability to avoid requesting family assistance in seeking Western medicine, and thus reaffirm caregiver roles [39]. Secondly, family history was a key facilitator of the use of TCM. TCM strategies are derived from family traditions and can be passed down as family knowledge [40]. For example, one participant shared that their grandmother taught the whole family to rub salt on their teeth after meals, to prevent tooth infections [40].

Fourteen (58%, 14/24) studies found that community factors influence the use of TCM [22,23,24,25,27,29,34,36,37,39,40,41,42,43]. These include culture, social, religion, residency in North America, and other factors. Firstly, a person’s country of origin, China, is a significant predictor of using TCM [34,37]. Immigrants from China use more TCM in comparison to those from Hong Kong, Taiwan, and Southeast Asia [34]. Older Chinese individuals born in Canada reported a lower level of agreement compared to those from Hong Kong, emphasizing the importance of a Chinese-focused culturalization in using TCM [37]. Secondly, social factors impact the use of TCM. The practice of TCM involves self-learning and teaching others; American and Canadian seniors often give each other advice on health [22,25,27,29,34,39]. TCM fulfills social roles for Chinese seniors, and the possibility of TCM use increased as seniors obtained more social support [34,39]. Thirdly, religion is a significant contributor to the use of TCM. Many Chinese health beliefs are rooted in Buddhism and Confucianism [37]. Canadian individuals who engage in ancestor worship and Buddhism are more likely to engage in traditional care [23]. Fourthly, the location and length of residency in North America can impact the use of TCM. Immigrants who have shorter residencies in the USA reported a higher use of TCM in comparison to their counterparts [41]. About the location of residency, Chinese elders in Vancouver are more likely to see a TCM practitioner compared to Chinese seniors in other Canadian cities [34].

Other factors include cost and transportation. Cost is a controversial factor in TCM. A USA qualitative study found cost to be a barrier to TCM, as the therapies were not covered under Medicaid [29]. However, a USA cross-sectional study reported that seniors of lower income use TCM more than those with higher income [41]. Transportation can be a barrier or facilitator [29]. For individuals who live in a Chinese-centric area, such as Chinatown, transportation is a facilitator, as the TCM practitioners are nearby [29]. For individuals who live further away, transportation is a barrier [29].

## 4. Discussion

### 4.1. Summary of Findings

In the majority of included studies, over half of Chinese North American seniors used a form of TCM. The most popular form of TCM was over-the-counter herbal products. Using TCM is an integral aspect of older Chinese adults’ cultural practices. Chinese seniors used various types of TCM therapies, including over-the-counter herbal products, TCM-based physical activities, consultations with TCM practitioners, and TCM food practices and therapies. Use of TCM among older Chinese adults was related to musculoskeletal symptoms, cardiovascular symptoms, SARS prevention, cancer screening, and oral health. Chinese seniors integrate TCM with Western medicine in their healthcare practices. Individual, family, and community factors influenced older Chinese adults’ use of TCM. Individual factors included physical health and mental health. Family factors included filial piety and family history. Community factors included culture, social, religion, residency in North America, and other factors.

### 4.2. Experiences

#### 4.2.1. Prevalence

Our review indicates a high prevalence of TCM use among Chinese seniors in North America. This finding is consistent with existing evidence. Compared to other ethnicities, Asian American seniors have the highest prevalence of alternative medicine usage, followed by Hispanic, African American, and Caucasian American older adults. Asian American seniors are 2.76 times (95% CI = 1.58–4.81) more likely to use biologically based therapies compared to older Caucasian Americans and are twice as likely to believe in mind-manipulation techniques to influence bodily health [45]. About 60% of Chinese Canadian adults have used some form of alternative medicine within the past year, and many used TCM in combination with Western medicine. Similar to our findings in seniors, Chinese Canadians are significantly more likely to use herbal therapy and acupuncture compared to Caucasian Canadians [46]. In comparison with studies on Chinese North American adults, Chinese seniors appear to have similar patterns of alternative medicine usage. Therefore, as the population of Chinese seniors increases in North America, there may be a greater number of individuals seeking TCM therapies. Due to the limited total sample size of included papers in this review, it was difficult to estimate the true prevalence rate among Chinese seniors in North America. Thus, in community practice and research, it is important to examine the local status before designing and implementing any related interventions. Future studies of large sample sizes, such as government-funded community surveys, are needed to describe the prevalence of TCM use.

#### 4.2.2. Use TCM in Various Illnesses and Potential Outcomes

Findings of our review suggest that Chinese seniors in Canada and the USA use various types of TCM therapies to prevent and treat different health conditions. However, research investigating the effectiveness of the related interventions is sparse. Since our review reveals that many Chinese North American seniors prefer TCM over Western therapies, future research should focus on incorporating evidence-based TCM interventions into practice. For example, several pilot studies in which nurses provided educational materials on Tai Chi exercises and TCM food therapy to Chinese patients with hypertension reported promising results [47,48]. In addition, future studies should also consider the ethnicity of their participants, recruitment methods, and definition of their measures of TCM. It is also important to note that studies on Asian American seniors may not be generalized to Chinese American seniors due to unique differences in culture [41]. In addition, researchers may consider recruiting participants from TCM practices or Chinese community centers, as there may be a substantial number of Chinese seniors who do not visit Western healthcare facilities [41]. TCM encompasses a broad collection of different treatments, and future studies should be careful to define which TCM practices to examine. Lastly, given the heterogeneity of study designs, TCM modalities, and outcome measures across the included studies, this scoping review did not aim to synthesize specific outcomes; future research, such as a systematic review with more narrowly defined inclusion criteria, is warranted to comprehensively evaluate the outcomes associated with TCM use among Chinese seniors.

#### 4.2.3. Integration

Our findings indicate that many Chinese seniors use TCM and Western medicine concurrently. This pattern is consistent with existing literature on Chinese populations. For example, a high proportion of mainland Chinese women with breast cancer report using complementary or alternative therapies alongside Western treatments [49]. Similarly, Chinese seniors in North America may engage in concurrent use, particularly during serious illnesses. However, such concurrent use raises important safety concerns. Certain TCM herbal therapies may interact with prescribed medications, and older adults may be at increased risk of adverse herb–drug interactions [50]. These risks may be further compounded by underreporting, as some seniors may be reluctant to disclose TCM usage due to language barriers [29,40]. Non-disclosure may also reflect broader systemic and relational factors, including fear of judgment, prior experiences of discrimination, or perceptions that healthcare providers may not understand or value TCM practices [37,39]. Such dynamics highlight underlying power imbalances within healthcare encounters, which may limit open communication and trust. Therefore, healthcare providers should proactively inquire about TCM usage when assessing and managing care [14]. In addition, fostering culturally safe and respectful care environments is essential to support disclosure and promote more effective integration of TCM and Western medicine.

Our findings also suggest that integration between TCM and Western medicine remains limited and complex. Evidence indicates that TCM usage may, in some cases, be associated with lower adherence to prescribed Western medical treatments or delays in seeking biomedical care [30,35,37]. For example, Chinese seniors may prefer TCM as a first-line approach and turn to Western medicine only when symptoms persist or worsen, particularly for acute conditions. Such patterns may contribute to delayed engagement in preventive services, including disease screening [27]. These behaviors are closely linked to cultural health beliefs and preferences, including perceptions of TCM as more holistic, natural, and aligned with traditional understandings of health and illness [37,39]. At the same time, structural barriers such as limited culturally appropriate services and fragmented communication between TCM and Western healthcare providers may further hinder effective integration [29]. Despite these challenges, emerging evidence suggests that integrative and collaborative approaches may improve health service utilization. For instance, one pilot study demonstrated that when TCM practitioners provided culturally relevant education on colorectal cancer screening, participants were more likely to complete screening compared to control groups [51]. This finding highlights the potential value of involving TCM practitioners in culturally tailored health promotion strategies. Overall, strengthening collaboration between TCM and Western healthcare providers, as well as improving culturally responsive care, may support better integration and enhance engagement with preventive and therapeutic services among Chinese seniors.

Future research on TCM usage among older Chinese adults should further examine how to effectively integrate TCM and Western health practices within culturally and structurally responsive care models. Building on the challenges identified in this review—including concurrent use, communication barriers, and limited coordination between providers—future studies should move beyond describing patterns of usage to developing and evaluating integrative care approaches. Such integration has the potential to strengthen patient–provider relationships, enhance trust, and improve treatment adherence [16]. In particular, improving healthcare providers’ cultural competence and understanding of TCM practices may facilitate more open communication, increase disclosure of TCM use, and promote timely engagement in preventive services such as disease screening. These efforts may also help address systemic barriers identified in this review, including differing health beliefs and perceived lack of provider understanding [37,39]. In addition, greater attention to patient safety is needed, especially regarding the concurrent use of TCM herbal therapies and Western medications. Education for both healthcare providers and patients is essential to reduce the risk of herb–drug interactions and contraindications [3,13,14,50]. Overall, a deeper understanding of the cultural, relational, and structural factors influencing TCM use, along with the development of collaborative and culturally safe care strategies, may support more effective and integrated healthcare delivery for Chinese seniors in North America.

### 4.3. Influencing Factors

Our findings suggest that individual factors, such as physical health, mental health, education level, gender, and other factors, can influence TCM usage. In support of our findings, a retrospective analysis of 5750 Americans reports that higher education and poorer physical health, such as co-morbidities, pain duration, pain severity, and functional limitations, are associated with greater complementary and alternative medicine use [52]. Additionally, a systematic review of 27 articles supports our findings that women are prominent in the field of complementary medicine and holistic spirituality [53]. The systematic review concluded that women experience personal empowerment that stems from power from within [53]. Through descriptive analysis of 10,233 children in the USA, mental health is identified as a facilitator of complementary and alternative medicine [54]. Herbal remedies, mind–body therapies, and chiropractic care are the most frequently used treatments [54]. The primary reasons for children to use complementary and alternative medicine include that it is natural, holistic, and helpful [54]. This further supports mental health as an influence of TCM and explains the conflicting findings of our results. Further research is needed to determine significant individual factors influencing TCM use and to strategize TCM accessibility for those in need.

The health behaviors of other ethnic groups in North America are also influenced by familial factors, similar to our findings in Chinese seniors. A cross-sectional study reports that in Caucasian American adult populations, recommendations from family influence their choices to use herbal therapy, acupuncture, or massage therapy [46]. A national telephone survey of 3172 women reports that Chinese, Mexican, and African American women’s choice to use alternative medicine is most influenced by family, whereas Caucasian women are more influenced by their physicians and media sources [55]. Considering these influences on healthcare decisions, providers should ensure that patients are provided with culturally sensitive support in decision-making, which should also involve family consultation.

Our findings suggest that the use of TCM among Chinese seniors is not only a health practice but also an important expression of cultural identity and continuity. TCM usage reflects deeply embedded beliefs about health, balance, and the body, and serves as a means of maintaining connections to cultural traditions, particularly in immigrant contexts [37,39]. Similarly, a scoping review of 126 studies on traditional Indigenous medicine in North America finds that such practices support connections to ancestral knowledge, cultural identity, and community belonging [56]. Beyond these similarities, both TCM and Indigenous medicine can be understood within a broader sociocultural context as practices that coexist with, and at times challenge, dominant biomedical models. In North American healthcare systems, which are largely grounded in Western biomedical paradigms, the continued use of traditional healing practices may also reflect efforts to preserve cultural knowledge and assert alternative understandings of health and well-being. These dynamics highlight the importance of recognizing traditional medicine not only as complementary therapies but also as culturally meaningful practices shaped by historical, social, and structural factors. In addition, from a public health perspective, these findings underscore the importance of culturally sensitive and culturally safe care in supporting diverse patient populations. Integrating cultural practices into healthcare delivery may enhance patient engagement, trust, and health outcomes [57]. For example, a pilot intervention, Chinese Medicine as Longevity Modality, demonstrated that culturally tailored approaches were well received among Chinese seniors and supported health engagement [47]. Future research should further explore how culturally grounded health interventions can be designed to respect and incorporate traditional practices while ensuring safety and effectiveness. Such approaches may contribute to more equitable and responsive healthcare systems for culturally diverse populations.

Our findings suggest that a small proportion of the Chinese Canadian sample of immigrants used TCM alone, which could be attributed to the fact that Western healthcare is government-funded, while TCM is not [34]. The article further speculates that individuals with limited finances are more likely to use Western care, regardless of personal preference, because of insurance coverage [34]. Similar to our findings in Chinese seniors, the high cost of some Western medications encourages 27.3% of Mexican Americans and 16.8% of African Americans to use alternative medicine instead of conventional therapies [55]. Individuals with lower income, including some Chinese seniors, may be more likely to seek alternative medicine if it is the more affordable option. Although a significant proportion of Chinese seniors may use TCM due to low income, only 5.7% of Chinese adults reported the cost of Western medication as an influencing factor [55]. Thus, the cost of Western medication may influence TCM usage in Chinese seniors more than Chinese adults, who are working and likely more financially stable. Future steps can be taken to explore the financial possibility of subsidizing TCM care within government health coverage.

### 4.4. Implications

First, healthcare providers should establish an understanding of TCM for safety reasons and for awareness of the health, social, cultural, and moral reasoning behind its usage among the Chinese senior immigrant population [58]. As some TCM herbal therapies may be effective in preventing diseases that are common among senior populations [33], healthcare providers and organizations should explore strategies to include TCM in current healthcare services. TCM herbal products may cause adverse drug interactions with Western therapies, and thus, healthcare providers need to be competent in discussing TCM usage with elders [50].

Second, further research is necessary to determine how institutions can provide the correct training and education to healthcare providers regarding TCM [16]. As well, the application of evidence-based medicine to TCM is needed to improve knowledge translation among Western healthcare providers [58]. The best research approach would be to start with evaluating the safety and efficacy of TCM products in humans by research trials, and then move on to mechanism and active substance studies in the identified efficacious TCM products [58].

Third, policymakers should examine the safety and efficacy of TCM products and consider licensing requirements for these products. These licensing requirements should ensure that providers have a strong knowledge of the potential interactions between Western medicines and TCM to protect individuals [34]. In addition, TCM can also be integrated into public health practices. TCM could be utilized by public health agencies to engage minority populations in disease prevention by providing culturally appropriate means to maintain their health.

### 4.5. Limitations

There are some limitations in this review. Firstly, the inclusion of studies in which at least 50% of participants were aged 65 years or older may have introduced heterogeneity, as some findings could reflect the experiences of middle-aged individuals. This should be considered when interpreting the results. Secondly, due to the limited number of articles on the use of TCM in minority populations, studies from both Canada and the USA were included in the review to give a more holistic summary of TCM use in North America. However, the differences between the experiences of Chinese individuals living in these countries differ, as do the healthcare systems. Thirdly, Canada and the USA span a large variety of settings, including rural, suburban, and urban. The setting, and thus access to TCM, could have an impact on the seniors’ use of TCM. Further research should specifically address the different settings in Canada and the USA and investigate the impact on TCM use. Fourthly, this review may be subject to publication bias, as only English-language, peer-reviewed studies were included. Studies with non-significant or less favorable findings may be underrepresented in the published literature. In addition, relevant studies published in Chinese or in gray literature may have been excluded, which may influence the comprehensiveness and interpretation of the findings. Lastly, this review was not prospectively registered (e.g., in PROSPERO), which may limit transparency and introduce potential risk of bias related to post hoc decision-making. However, a predefined methodological approach was followed, including clearly specified inclusion criteria and systematic procedures, to mitigate this risk.

## 5. Conclusions

This scoping review summarizes the experiences of Chinese senior immigrants in Canada and the USA in using TCM. The factors that influence TCM use were also identified, including individual, familial, and community factors. Throughout the included studies, seniors used a variety of TCM, ranging from self-administered to professionally prescribed, including TCM food practices and therapies, TCM-based physical activities, over-the-counter herbal products, and TCM practitioner consulting. This review suggested that Chinese seniors frequently use TCM in routine maintenance of their health and prevention of diseases. It is important for healthcare providers and policymakers to understand TCM and to be able to provide the best quality of care for Chinese seniors in Canada and the USA. Future research should be conducted in various settings in Canada and the USA, including rural, suburban, and urban, to better understand the use of TCM in different populations.

## Figures and Tables

**Table 1:** Characteristics of included studies.

	Authors (Year) Research Setting	Research Purpose and Methodology	Sample Characteristics	Types of TCM
1	Allison (1993) [22] Southwestern Metropolitan City, USA; Community	To identify and analyze the activity patterns and the nature of leisure among elderly Chinese Americans. QL: interviews	25 older Chinese Americans (10 M, 15F), aged 64 to 85	Tai Chi
2	Chappell (1998) [23] Vancouver and Victoria, British Columbia, Canada; Community	To assess whether the same factors are predictive of health service utilization among Chinese elders as is true of the general population of seniors. QN: cross-sectional study	602 Chinese elders (213 M, 389 F)	Food therapy, TCM self-use, TCM consult, acupuncture, bone setters
3	Lew-Ting (1998) [24] Los Angeles, California, USA, Taipei, Taiwan; Community	To report ethnographic and statistical data on the indigenous Chinese concept of constitution (Ti-Zhi) and its association with health status. QL: interview	203 Chinese elderly (98 M, 105 F), aged at least 59	Constitution, Hot/cold Dichotomy
4	Ren (1998) [12] Boston, Massachusetts, USA; Community	To explore how elderly Chinese perceive their own health. QN: surveys	219 elderly Chinese (84 M, 135 F), average age 69	Not reported
5	Torsch (2000) [25] Houston, Texas, and Philadelphia, Pennsylvania, USA; Community	To compare the health perceptions, concerns, and coping strategies among Chinese elders in two Asian and Pacific Islander American communities. QL: ethnographic interviews	63 Chinese elders aged at least 55; 58 Chamorro aged at least 55	Herbal medicines and teas, TCM exercises
6	Tjam (2002) [26] Kitchener, Waterloo Region, Ontario, Canada; Community	To explore the health, psychosocial, and cultural determinants of using TCM and Western medicines among Chinese-Canadian older persons. QN: cross-sectional	106 elderly Chinese, majority between 65 and 74 years; 39 M, 67 F	Over-the-counter TCM
7	Pang (2003) [27] Los Angeles, California, USA; Community	To analyze the health-seeking behaviors of community-dwelling elderly Chinese Americans on the influences of family network, cultural values, and immigrant experience in their use of health resources. QL: Focus group interviews	25 elderly Chinese Americans (11 M, 14 F), average age 72	Acupuncture, herbal medicine, Tai Chi, exercise, nutritional diet, home remedies
8	Wu (2004) [28] Boston, Massachusetts, USA; Community	To explore the unique effects of various chronic illnesses on depression in a sample of Chinese immigrant elders. QN: cross-sectional	177 older Chinese Americans (67 M, 110 F), average age 71.8 years	TCM, detailed type not reported
9	Aroian (2005) [29] Boston, Massachusetts, USA; Community	To explore the patterns and reasons for health and social service use among Chinese immigrant elders. QL: interviews	27 Chinese immigrant elders (10 M, 17 F); 11 family caregivers (4 M, 7 F); 12 care professionals	Acupuncture and herbal medicine
10	Li (2006) [30] San Francisco, California, USA; Clinic	To characterize Chinese immigrants with hypertension and to examine what cultural factors are associated with medication adherence. QN: cross-sectional	200 Chinese immigrants (100 M, 100 F), mean age 71 years	Chinese herbs, visiting Chinese medicine professionals
11	Taylor-Piliae (2006) *** [31] San Francisco, California, USA; Community	To examine the change in psychosocial status following a 12-week Tai Chi exercise intervention among ethnic Chinese people with cardiovascular disease risk QN: quasi-experimental	39 Chinese elders, average age of 66 years; 27 women, 12 men	Tai Chi
12	Taylor-Piliae (2006) *** [32] San Francisco, California, USA; Community	To determine whether Tai Chi improves balance, muscular strength, endurance, and flexibility over time. QN: quasi-experimental	39 Chinese elders, average age of 66 years; 27 women, 12 men	Tai Chi
13	Taylor-Piliae (2006) *** [33] San Francisco, California, USA; Community	To determine the hemodynamic responses to a 12-week community-based Tai Chi exercise intervention among ethnic Chinese with cardiovascular disease risk QN: quasi-experimental	39 Chinese elders, average age of 66 years; 27 women, 12 men	Tai Chi
14	Lai (2007) ** [34] 7 Canadian cities (Victoria, Calgary, Toronto, Montreal, etc.), Canada; Community	To understand the prevalence and predictors of TCM use by older Chinese immigrants in Canada QN: cross-sectional	2167 elderly Chinese immigrants, average age 69.8 years; 943 M, 1224 F	Chinese herbs, acupuncture, bone setter, moxibustion, Qi Qong, etc.
15	Li (2008) [35] San Francisco, California, USA; Community	To explore the relationship between demographic and cultural factors and antihypertensive medication adherence in older Chinese immigrants. QN: cross-sectional	144 older Chinese immigrants (75 M, 69 F), average age 75.2 years;	Chinese herbs
16	Wills (2008) [36] Edmonton, Alberta, Canada; Community	To describe the responses of the Chinese elderly during the severe acute respiratory syndrome pandemic, and their use of Western medicine and TCM Mixed Methodology: QN: constant comparison; QL: interviews	19 Chinese elders (6 M, 13 F), aged from 65 to 90; 4 TCM practitioners	Herbs, special foods, Tai Chi, Qi Qong
17	Lai (2009) ** [37] 7 Canadian cities (Victoria, Calgary, Toronto, Montreal, etc.), Canada; Community	To examine the cultural health beliefs held by Chinese elders in Canada. QN: cross-sectional	2272 older Chinese (934 M, 1338 F), average age 69.8 years	Herb and healthy diets
18	Wang (2010) [38] Pittsburgh, Pennsylvania, USA; Community	To explore the perceptions and self-management practices of Chinese elders regarding treatment adherence, lifestyle decisions, and patient-provider communication within the context of their culture. QL: focus groups	19 older Chinese Americans (9 M, 10 F), aged at least 65	Danshen root, sanchi, or integripetal rhodiola herb
19	Kong (2012) [39] Oklahoma City, Oklahoma, USA; Community	To explore how TCM is used as a tool, a resource, and a product of meaning-construction in elderly Chinese immigrants’ everyday life. QL: interviews	20 elderly Chinese immigrants (8 M, 12F), average age 65	Herbal product, healthy diets
20	MacEntee (2012) [40] Vancouver, Canada, and Melbourne, Victoria, Australia; Community	To explore how elderly Chinese immigrants value and relate to how acculturation influences oral health and subsequent service use. QL: focus groups	28 older Chinese immigrants (34 M, 17 F), aged 65 years or higher	Herbal teas, using salt water to rinse teeth after eating
21	Dong (2015) * [41] Chicago, Illinois, USA; Community	To provide an estimate of TCM use among Chinese older adults in the USA and to examine associations between sociodemographic characteristics, health measures, and TCM use. QN: cross-sectional	3158 older Chinese Americans (1298 M, 1858 F), average age 72.8	Acupuncture, massage, herbs, Tai Chi
22	Dong (2018) * [42] Chicago, Illinois, USA; Community	To examine the association between musculoskeletal symptoms and different subtypes of TCM usage. QN: cross-sectional	3157 older Chinese Americans (1298 M, 1859 F), average age 72.8	Eight subtypes of TCM: herbal products, acupuncture, Tai Chi, etc.
23	Dong (2018) * [43] Chicago, Illinois, USA; Community	To examine the association between their cancer screening behaviors and self-reported cancers with TCM use. QN: cross-sectional	1830 older female Chinese Americans, aged 60–75	Tai Chi, herbal remedies, and massage therapy
24	Chao (2020) * [44] Chicago, Illinois, USA; Community	To examine the association between anxiety symptoms, depressive symptoms, and TCM use among United States Chinese older adults. QN: cross-sectional	3157 older Chinese Americans (1298 M, 1859 F), average age 72.8	Eight subtypes of TCM: herbal products, acupuncture, Tai Chi, etc.

Notes: TCM = Traditional Chinese Medicine; M = males; F = females; QL = qualitative; QN = quantitative; USA = United States of America. Studies marked with the same symbol represent analyses derived from the same underlying study population. In calculating the total sample size, each dataset was counted only once to avoid duplication. When multiple studies using the same dataset reported different sample sizes, the largest reported sample was used. Specifically, * indicates the Chicago cohort (Dong 2015 [41]; Dong 2018 [42]; Dong 2018 [43]; Chao 2020 [44]; largest n = 3158), ** indicates the Canadian cohort (Lai 2007 [34]; Lai 2009 [37]; largest n = 2272), and *** indicates the San Francisco Tai Chi intervention cohort (Taylor-Piliae et al. [31,32,33], 2006; n = 39). The total unique sample size across the included studies was 7346 participants after accounting for overlapping study populations. Gender distribution was variably reported across studies and was therefore not aggregated.

## Data Availability

The data supporting the findings of this study are available in the Supplementary Materials. Further inquiries can be directed to the corresponding authors.

## Supplementary Materials

The following supporting information can be downloaded at: https://www.mdpi.com/article/10.3390/healthcare14101310/s1, Table S1: Quality assessment of qualitative studies; Table S2: Quality assessment of cohort study; Table S3: Quality assessment of observational cohort and cross-sectional studies.
