# Supplementary material for: The Use of Traditional Chinese Medicine Among Chinese Seniors in Canada and the United States: A Scoping Review
Source: Healthcare (Basel). 2026 May 12;14(10):1310. doi: 10.3390/healthcare14101310 (PMC13205178; doi:10.3390/healthcare14101310)
Supplement: Supplementary file 1 [file healthcare-14-01310-s001.zip › healthcare-4218121-supplementary.pdf]

Summary of the quality assessment:
The Critical Appraisal Skills Program (CASP) Checklists have been applied as quality assessment tools to assess the included qualitative methodology papers. For the cross-sectional studies, the National Institutes of Health (NIH) Quality Assessment Tool for Observational Cohort and Cross-Sectional Studies was used. Using these checklists and consulting current literature in the field, we classified the quality of papers as low, moderate, or high according to the final score, for the CASP Checklists, or as good, fair, or poor, for the NIH Tool. Different researchers independently evaluated each article and triangulated the final scores. All papers included in this review were of moderate/fair to high/good quality.

Supplemental Table S1: Quality assessment of qualitative studies

Paper	Q1	Q2	Q3	Q4	Q5	Q6	Q7	Q8	Q9	Q10	Total Score (out of 10); Level of evidence (high/medium/low)
Allison (1993) [22]	Yes	Yes	Yes	Yes	Yes	Yes	Yes	Yes	No	Yes	9; High
Aroian (2005) [29]	Yes	Yes	Yes	Yes	Yes	No	Yes	Yes	Yes	Yes	9; High
Chappell (1998) [23]	Yes	Yes	Yes	Yes	Yes	No	Yes	Yes	Yes	Yes	9; High
Kong (2012) [39]	Yes	Yes	Yes	No	Yes	Yes	Yes	Yes	Yes	Yes	9; High
Lew-Ting (1998) [24]	Yes	Yes	Yes	Yes	Yes	No	No	Yes	Yes	Yes	8; Medium
MacEntee (2012) [40]	Yes	Yes	Yes	Yes	Yes	No	Yes	Yes	Yes	Yes	9; High
Pang (2003) [27]	Yes	Yes	Yes	Yes	Yes	No	Yes	Yes	Yes	Yes	9; High
Torsch (2000) [25]	Yes	Yes	Yes	Yes	Yes	No	No	Yes	Yes	Yes	8; Medium

Wang (2010) [38]	Yes	Yes	Yes	Yes	Yes	No	No	Yes	Yes	Yes	8; Medium
Wills (2008) [36]	Yes	Yes	Yes	Yes	Yes	Yes	Yes	Yes	Yes	Yes	10; High
Total of YES	10	10	10	10	10	2	6	10	9	10	

Notes:
10-point scale: high = greater than 8, moderate = 7-8, low = 6 or less

Q1: Did the trial address a clearly focused issue?
Q2: Is a qualitative methodology appropriate?
Q3: Was the research design appropriate to address the aims of the research?
Q4: Was the recruitment strategy appropriate?
Q5: Was the research design appropriate to address the aims of the research?
Q6: Has the relationship between researcher and participants been adequately considered?
Q7: Have ethical issues been taken into consideration?
Q8: Was the data analysis sufficiently rigorous?
Q9: Is there a clear statement of findings?
Q10: Is the research valuable?

Supplemental Table S2: Quality assessment of cohort study

Paper	Q1	Q2	Q3	Q4	Q5	Q6	Q7	Q8	Q9	Q10	Q11	Q12	Total Score (out of 12); Level of evidence (high/medium/low)
Taylor-Piliae (2006) [31]	Yes	Yes	Yes	Yes	No	Yes	Yes	Yes	Yes	Yes	Yes	Yes	11; high
Taylor-Piliae (2006) [32]	Yes	Yes	Yes	Yes	No	Yes	Yes	Yes	Yes	Yes	Yes	Yes	11; high
Taylor-Piliae (2006) [33]	Yes	Yes	Yes	Yes	No	Yes	Yes	Yes	Yes	Yes	Yes	Yes	11; high
Total of YES	1	1	1	1	0	1	1	1	1	1	1	1	

Notes:
12-point scale: high = greater than 10, moderate = 9-10, low = 8 or less

Q1: Did the trial address a clearly focused issue?
Q2: Was the cohort recruited in an acceptable way?
Q3: Was the exposure accurately measured to minimise bias?
Q4: Was the outcome accurately measured to minimise bias?
Q5: Are cofounding factors identified and taken into consideration in design/analysis?
Q6: Is follow-up long and thorough?
Q7: What is the strength of the results?
Q8: How precise are the results?
Q9: Are the results believable?
Q10: Can the results be applied in your context? (Or to the local population)
Q11: Do the results of this study fit with other available evidence?
Q12: What are the implications of this study for practice?

Supplemental Table S3: Quality assessment of observational cohort and **cross-sectional studies**

Paper	Q1	Q2	Q3	Q4	Q5	Q6	Q7	Q8	Q9	Q10	Q11	Q12	Q13	Q14	Quality Rating (Good, Fair, Poor)
Chao (2020) [44]	Yes	Yes	Yes	Yes	No	NA	NA	NA	NA	NA	Yes	NA	NA	NA	Good
Dong (2015) [41]	Yes	Yes	Yes	Yes	No	NA	NA	NA	NA	NA	Yes	NA	NA	NA	Good
Dong (2018) [42]	Yes	Yes	Yes	Yes	No	NA	NA	NA	NA	NA	Yes	NA	NA	NA	Good
Lai (2007) [34]	Yes	Yes	Yes	Yes	Yes	NA	NA	NA	NA	NA	Yes	NA	NA	NA	Good
Lai (2009) [37]	Yes	Yes	Yes	Yes	No	NA	NA	NA	NA	NA	Yes	NA	NA	NA	Good
Ren (1998) [12]	Yes	Yes	Yes	Yes	No	NA	NA	NA	NA	NA	Yes	NA	NA	NA	Good
Tjam (2002) [26]	Yes	Yes	Yes	Yes	Yes	NA	NA	NA	NA	NA	Yes	NA	NA	NA	Good
Li (2006) [30]	Yes	Yes	Yes	Yes	No	NA	NA	NA	NA	NA	Yes	NA	NA	NA	Good
Li(2008) [35]	Yes	Yes	Yes	Yes	Yes	NA	NA	NA	NA	NA	Yes	NA	NA	NA	Good
Dong (2018) [43]	Yes	Yes	Yes	Yes	No	NA	NA	NA	NA	NA	Yes	NA	NA	NA	Good
Wu (2004) [28]	Yes	Yes	Yes	Yes	No	NA	NA	NA	NA	NA	Yes	NA	NA	NA	Good
Total of YES	11	11	11	11	3	0	0	0	0	0	11	0	0	0	

Notes:
Overall quality rating is not done by summing scores and assigning a rating based on if the sum falls within a range, but rather by looking at all 14 questions holistically and determining the overall quality of the paper. Therefore, each study is assessed on a case-by-case basis.

Q1. Was the research question or objective in this paper clearly stated?
Q2. Was the study population clearly specified and defined?
Q3. Was the participation rate of eligible persons at least 50%?

Q4. Were all the subjects selected or recruited from the same or similar populations (including the same time period)? Were inclusion and exclusion criteria for being in the study prespecified and applied uniformly to all participants?
Q5. Was a sample size justification, power description, or variance and effect estimates provided?
Q6. For the analyses in this paper, were the exposure(s) of interest measured prior to the outcome(s) being measured?
Q7. Was the timeframe sufficient so that one could reasonably expect to see an association between exposure and outcome if it existed?
Q8. For exposures that can vary in amount or level, did the study examine different levels of the exposure as related to the outcome (e.g., categories of exposure, or exposure measured as continuous variable)?
Q9. Were the exposure measures (independent variables) clearly defined, valid, reliable, and implemented consistently across all study participants?
Q10. Was the exposure(s) assessed more than once over time?
Q11. Were the outcome measures (dependent variables) clearly defined, valid, reliable, and implemented consistently across all study participants?
Q12. Were the outcome assessors blinded to the exposure status of participants?
Q13. Was loss to follow-up after baseline 20% or less?
Q14. Were key potential confounding variables measured and adjusted statistically for their impact on the relationship between exposure(s) and outcome(s)?
